# U.S.A. trends in intrahepatic and bile duct cancers from 1999–2023

**DOI:** 10.3389/fgstr.2026.1681142

**Published:** 2026-04-29

**Authors:** Samuel W. Berg, Taylor Billion, Abubakar Tauseef

**Affiliations:** 1 Internal Medicine, Creighton University Department of Internal Medicine, Omaha, NE, United States; 2 Internal Medicine, Creighton University School of Medicine, Omaha, NE, United States

**Keywords:** ALD (alcoholic Liver Disease), bile duct cancer, cholangiocarcinoma, HCC, MASLD

## Abstract

Liver and intrahepatic bile duct malignancies are an increasing cause of mortality in the United States due unclear reasons over the past 20 years, perhaps proportionately due to overall increase in age of the US population and improvement of diagnostic/screening strategies. This study utilizing the Centers for Disease Control and Prevention Wide-Ranging Online Data for Epidemiologic Research (CDC WONDER) database, investigated trends of demographic (gender, race, age group) and geographical differences in liver and intrahepatic bile duct cancer related mortality. Average annual percentage changes were assessed using the CDC WONDER data base with respect to age-adjusted mortality rates (AAMR) and annual percentage change (APC). This study used the Joinpoint Regression Program to determine statistical significance of mortality trends between 1999 and 2023. During this study period (1999-2023), overall higher mortality rates were noted in the American Indian and Alaskan native population and a decrease in mortality in the Asian and Pacific Islander population, with the South and West regions having the highest AAMR. Furthermore, mortality rates demonstrated significantly greater trend in liver and intrahepatic bile duct cancers in males than in females during the entire study period. These results serve to inform on demographics that resources should be focused towards to make policy and encourage screening and diagnosing for at risk groups and regions.

## 1 Introduction

Liver and intrahepatic bile duct cancers represent 2.1% of all new cancer diagnoses and 4.9% of all cancer related deaths; with only a 21.7% reported 5-year relative survival rate for these cancers (Liver and Intrahepatic Bile Duct Cancer — Cancer Stat Facts). Liver cancer primarily arises from aberrant hepatocytes, the most common type being hepatocellular carcinoma. Hepatocellular carcinoma approaches 80% of all liver cancers, found more commonly in patients with preexisting liver disease such as chronic alcohol-related liver disease, chronic hepatitis viral infections, as well as in metabolic dysfunction-associated steatotic liver disease (MASLD) (1). Unfortunately, liver cancers are often diagnosed late in the disease process making treatment and eradication in the patient challenging, and often unsuccessful (2). Furthermore, bile duct cancers, specifically cholangiocarcinoma, arise from the bile duct tissue connecting the liver to the small intestine. Cholangiocarcinoma represents the second most common liver malignancy next to hepatocellular carcinoma and the most common type of bile duct cancer (3). Bile duct malignancies account for nearly 3% of all gastrointestinal cancers, with an increased incidence of intrahepatic bile duct cancers of 4.36% over the last decade (4). Often, intrahepatic bile duct malignancies are mistaken for metastatic disease from an unknown primary site (5). Specifically, intrahepatic cholangiocarcinoma is often mistaken for cancer of unknown primary (CUP) origin, and in fact accounts or 21% of CUP after ensuing molecular profiling (6). Verily, this makes diagnosing and treating for these specific types of cancers challenging with respect to screening and treatment strategies. Often a combination of endoscopic ultrasound (EUS), MRI Cholangiopancreatography (MRCP), PET/CT scans, endoscopic brush cytology, as well as through lab workups are needed to achieve a diagnosis and appropriate staging (7–9).

The high death rates from liver cancers and intrahepatic bile duct cancers, even in the era of modern medicine still reflect a challenge when considering screening and treatment at a population level. This paper utilizes a cohort strategy, using de-identified demographic data about mortality rates in the United States to provide an in-depth review of liver and intrahepatic bile duct cancer mortality. It attempts to address trends in mortality from the years 1999-2023, and possibly account for underlying factors driving these disparities between gender, race, ages, and regions of the country. It will use a database of de-identified date that includes the entire country and all states, the Center for Disease Control and Prevention’s Wide-ranging ONline Data for Epidemiologic Research (10). Ultimately this research article will account for obstacles in treatment strategies for managing liver and intrahepatic bile duct malignancies; while also offering insight into possible future projects about this very topic. It is our hope that highlighting these trends may offer improved comprehension of these types of cancers is fundamental to diagnosing, managing, treating, and ultimately reducing the impact of liver and intrahepatic bile duct cancers on the US population and healthcare system.

## 2 Methods

The Centers for Disease Control and Prevention Wide-ranging Online Data for Epidemiologic Research (CDC Wonder) was utilized to collect mortality rate data due to liver and intrahepatic bile duct malignancies as a multiple cause of death in the US from 1999 to 2023 using categories: overall trend, gender, race (e.g.; non-Hispanic (NH) White, Black, American Indian/Alaskan Native, Asian/Pacific Islander, and Hispanic), 10-year age groups (e.g.; 25 to 39, 40 to 54, 55 to 69, 70 to 84, 85+ years of age), and geographic region. CDC Wonder data serves as a data acquisition point for other studies evaluating gastrointestinal malignancy trends over the past 20+ years (11, 12). This type of study, combined with the CDC Wonder database being anonymous and de-identified data made this research project exempt from institutional review board approval. Liver and intrahepatic bile duct malignancies related mortality was identified using the International Classification of Diseases (ICD), 10th Revision, Clinical Modification codes C22 in patients ≥25 years of age. The decision to exclude mortalities rates for individuals <25 years of age reflected the rarity of these types of cancers in this age range; while including 25+ year old age range reflected proportionate increase in risk of mortality related to liver and intrahepatic bile duct cancers.

The Joinpoint program (Joinpoint version 4.9.0.0 available from National Cancer Institute, Bethesda, Maryland) was used to calculate trends, defined as average annual percent change (AAPC) and to identify disparities between groups (Kim jpoint) (13). All age-adjusted rates (AAMR) are reported per 100,000. Liver and intrahepatic bile duct cancer related crude and age-adjusted mortality rates were calculated; where crude mortality rates were designed via dividing liver and intrahepatic bile duct cancer related deaths by the corresponding United States population. Using the Monte Carlo permutation test, AAMRs were calculated for the line segments linking a Joinpoint; and annual percentage change (APC) with 95% confidence intervals (CIs) for the AAMRs were calculated for the line segments. For the entire study period (1999-2024), the weighted average annual percent change APCs were calculated and reported as AAPCs and corresponding 95% CIs to summarize the reported mortality trends for liver and intrahepatic bile duct malignancies. AAPCs slopes increasing or decreasing were noted as significantly different from zero using a 2-tailed t-test, and statistical significance was set at p ≤0.05.

## 3 Results

### 3.1 Overall

For this study period (1999-2023), there were 583,843 reported deaths related to liver and intrahepatic bile duct malignancies via the CDC WONDER Database (**Table 1**). Three statistically significant J-point year ranges were determined: 1999-2007, 2007-2013, and 20132023.

**Table 1:** Liver and IHBD cancer-related age-adjusted mortality rate per 100,000 people; overall and stratified by gender, 1999-2023.

Year	Overall	Male	Female
1999	7.73	11.19	5.02
2000	7.76	11.46	4.86
2001	7.88	11.66	4.93
2002	8.15	11.98	5.04
2003	8.3	12.33	5.12
2004	8.47	12.28	5.29
2005	8.69	12.88	5.28
2006	8.79	12.9	5.38
2007	8.89	13.17	5.36
2008	9.29	13.97	5.37
2009	9.62	14.37	5.67
2010	9.86	14.69	5.81
2011	10.19	15.14	5.96
2012	10.59	15.75	6.2
2013	10.8	16.02	6.35
2014	10.89	16.06	6.49
2015	11.11	16.37	6.58
2016	11.23	16.51	6.73
2017	11.31	16.62	6.79
2018	11.32	16.58	6.85
2019	11.27	16.48	6.8
2020	11.33	16.37	7.05
2021	11.61	16.43	7.44
2022	11.49	16.34	7.37
2023	11.89	16.76	7.73

**Figure 1:**
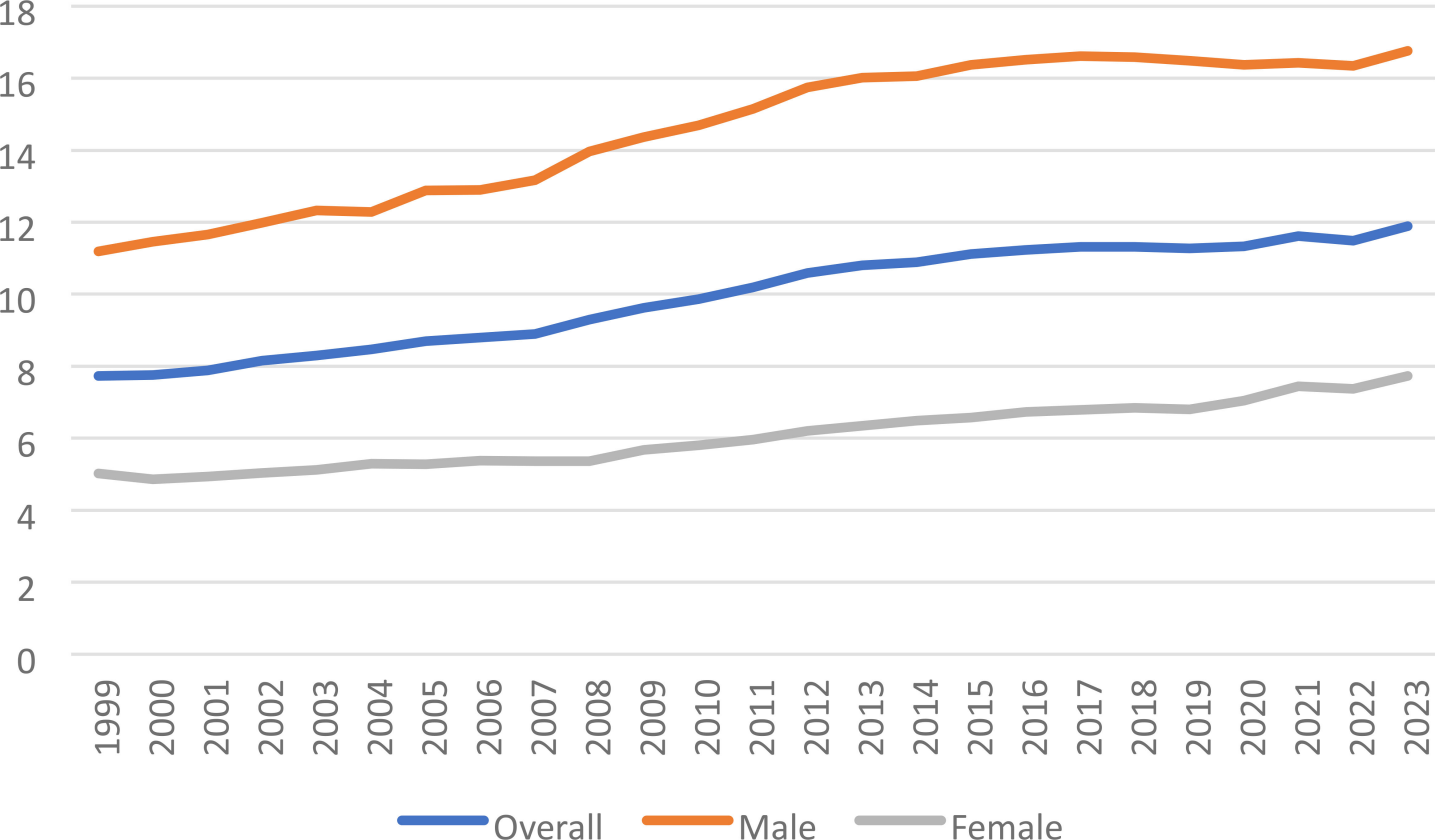
Liver and IHBD cancer-related age-adjusted mortality rate per 100,000 people; overall and stratified by sex, 1999-2023.

**Figure 2:**
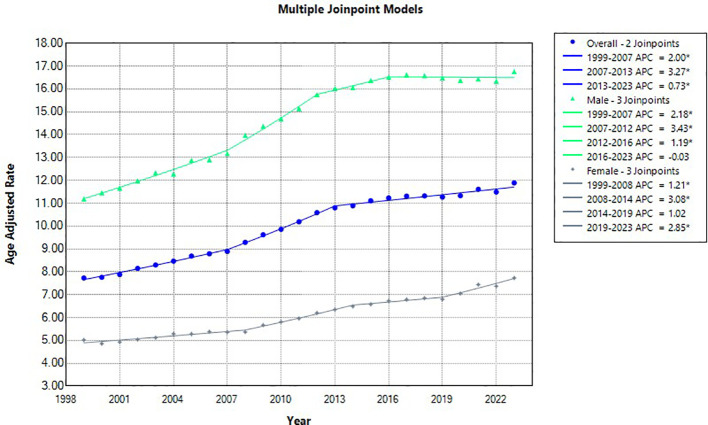
Joinpoint model of Liver and IHBD cancer-related AAMR per 100,000 people overall and stratified by sex, 1999-2023 (*indicates the APC is statistically significant).

Overall, the age-adjusted mortality rate (AAMR) increased from 7.73 (95% CI 7.76 to 7.86) in 1999 to 8.89 (95% CI 8.77 to 9.02) in 2007 with an APC of 1.99 (95% CI 1.09 to 2.34) (**Figures 1**, **2**). From 2007–2013 the age-adjusted mortality rate (AAMR) increased from 9.29 (95% CI 9.16 to 9.42) to 10.80 (95% CI 10.67 to 10.94), the APC in AAMR was 3.26* (95% CI 2.79 to 4.28). The AAMR increased from 2013-2023, 10.89 (95% CI 10.75 to 11.02) in 2013 to 11.89 (95% CI 11.76 to 12.02) in 2023, with an APC in AAMR then increasing significantly from 2014 to 2023 to 0.73* (95% CI 0.54 to 0.92). Overall AAPC from 1999–2023 revealed an AAPC of 1.78* (95% CI 1.70 to 1.87).

### 3.2 Gender

For males during this study period, there were 388,737 reported deaths related to liver and intrahepatic bile duct (IHBD) malignancies (**Table 1**). With respect to gender across the study range, the AAMR in men demonstrated 3 J-Point year ranges: 1999-2007, 2007-2012, 20122016, and 2016-2023; of those year ranges only 3 were significant in APC in AAMR: 19992007, 2007-2012, 2012-2016. The APC in AAMR for 1999–2007 was 2.18* (95% CI 0.99 to 2.73) from 2007-2012 3.43* (95% CI 1.85 to 4.46), and from 2012-2016 1.19* (95% CI 0.35 to 3.20) (**Figures 1**, **2**). Overall male AAPC from 1999–2023 reflected an AAPC of 1.62* (95% CI 1.52 to 1.73).

For females during this study period, there were 195,106 reported deaths related to liver and intrahepatic bile duct malignancies. In women, the AAMR demonstrated 3 J-Point years ranges: 1999-2008, 2008-2014, 2014-2019, and 2019-2023; only 3 of those year ranges were significant: 1999-2008, 2008-2014, and 2019-2023. The APC in AAMR for 1999–2008 was 1.20* (95% CI 0.49 to 1.62) from 2008-2014 3.08* (95% CI 2.41 to 4.53), and from 2019-2023 2.85* (95% CI 2.05 to 4.44) (**Figures 1**, **2**). Overall female AAPC from 1999–2023 demonstrated an AAPC of 1.90* (95% CI 1.79 to 2.02).

### 3.3 Race

**Figure 3:**
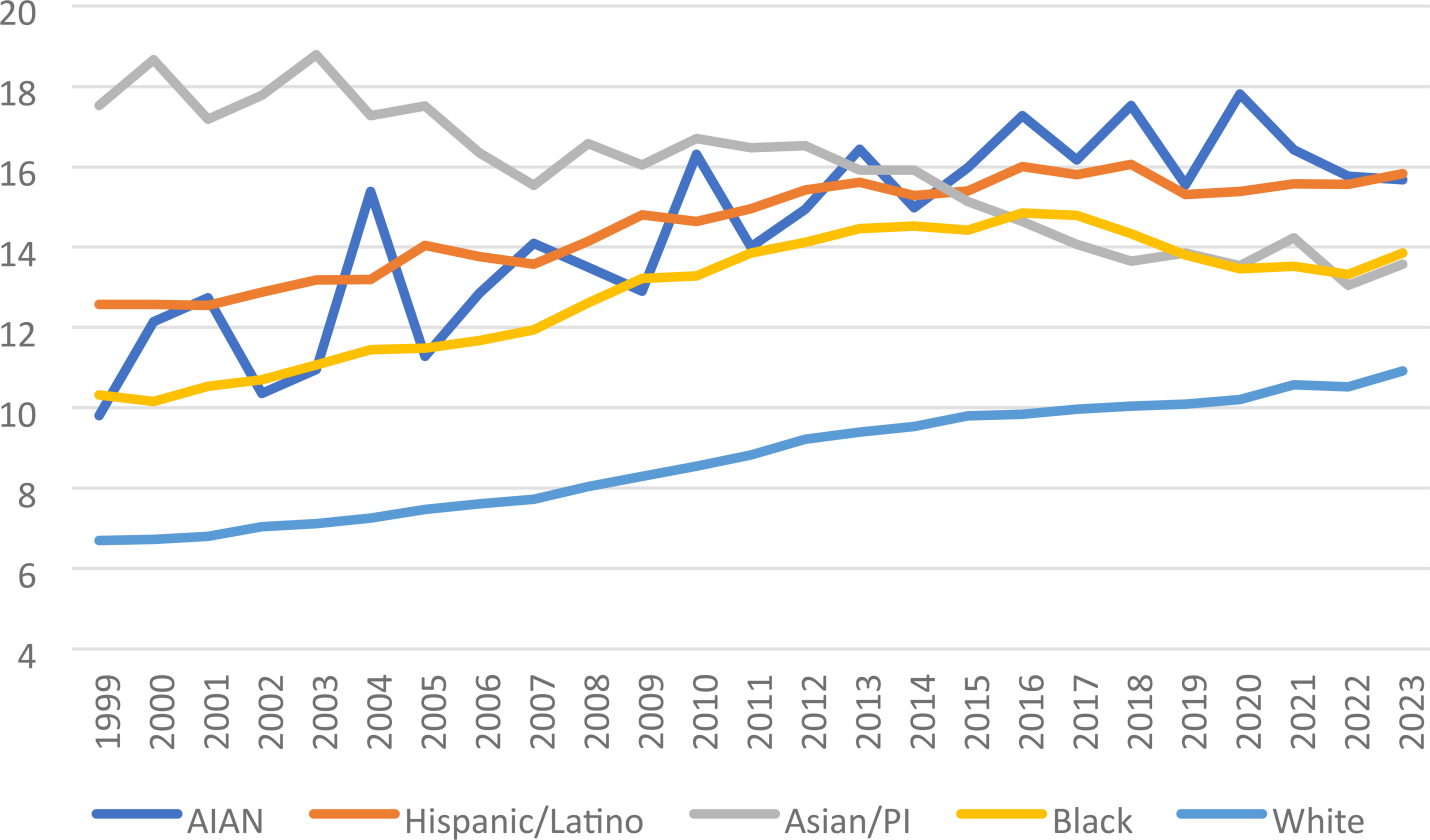
Liver and IHBD cancer-related age-adjusted mortality rate per 100,000 people; overall and stratified by race, 1999-2023.

**Figure 4:**
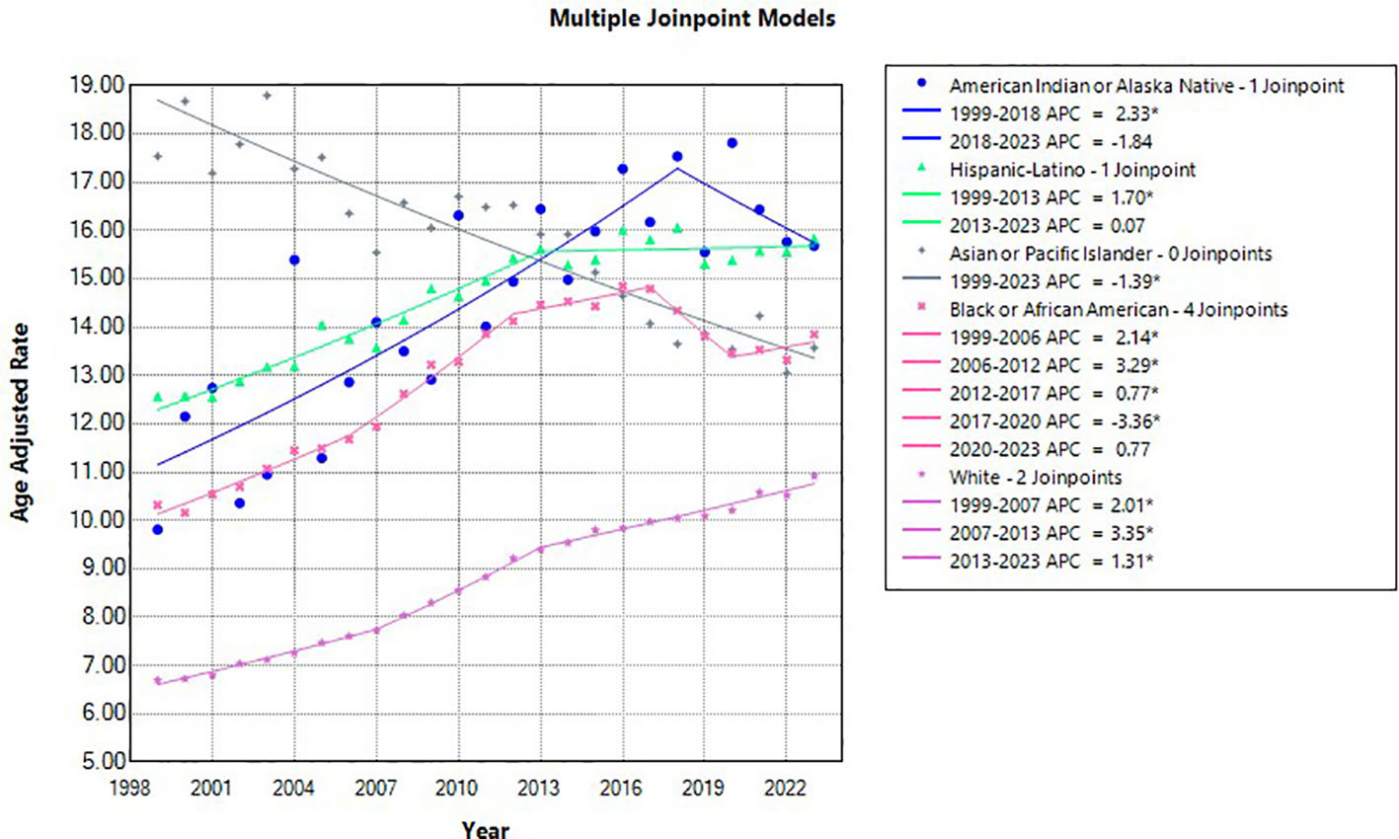
Joinpoint model of Liver and IHBD cancer-related AAMR per 100,000 people overall and stratified by race, 1999-2023 (*indicates the APC is statistically significant).

Liver and intrahepatic bile duct malignancies with respect to race demonstrated totals for the entire study (1999-2023) as follows: American Indian or Alaska native (AIAN) 5,117, AsianPacific Islander (Asian/PI) 36,714, Black or African 76,349, White non-Hispanic 391,606, and Hispanic-Latino 71,890 (**Table 2**). American Indian or Alaska Native demonstrated one J-point year range of significance, from 1999–2018 APC showed increased mortality: 2.33* (95% CI 1.69 to 16.49); AAPC for the entire study period demonstrated significance 1.44* (95% CI 0.56 to 3.47). Hispanic-Latino also only had one year range demonstrating significant increase in mortality, from 1999–2013 APC was 1.69* (95% CI 1.42 to 2.11); for the study period AAPC for Hispanic-Latino was significant at 1.01* (95% CI 0.88 to 1.18). While Asian or Pacific Islander had had the lowest mortality of all races, with significantly decreased APC for the entire study period (1999-2023), -1.39* (95% CI -1.65 to -1.06); the AAPC was also significantly decreased 1.39 (95% CI -1.65 to -1.06). Black or African race group had 3 significant year ranges with respect to APC and increased mortality: 1999-2006 2.13* (95% CI 0.53 to 2.58), 2006-2012 3.28* (95% CI 2.80 to 4.51), 2012-2017 0.76* (95% CI 0.18 to 1.47), and significantly decreased mortality APC in one range from 2017-2020 -3.35* (95% CI -4.08 to -2.19). For the entire study period, AAPC for Black or African race was significant at 1.26* (95% CI 1.14 to 1.37). The White non-Hispanic race category had three-year ranges J-point statistics concluded had increased mortality: 1999-2007 2.00* (95% CI 1.43 to 2.31), 2007-2013 3.35* (95% CI 2.92 to 4.27), and 2013-2023 (95% CI 1.13 to 1.47). With the AAPC for the entire study in White non-Hispanics being significant at 2.05* (95% CI 1.97 to 2.12). (**Figures 3**, **4**).

**Table 2:** Liver and IHBD cancer-related age-adjusted mortality rate per 100,000 people stratified by race, 1999-2023.

Year	AIAN	Hispanic/Latino	Asian/PI	Black	White
1999	9.81	12.57	17.53	10.32	6.7
2000	12.15	12.58	18.67	10.16	6.73
2001	12.74	12.55	17.18	10.54	6.8
2002	10.36	12.88	17.78	10.7	7.04
2003	10.95	13.18	18.79	11.07	7.12
2004	15.39	13.19	17.27	11.45	7.26
2005	11.29	14.05	17.51	11.49	7.47
2006	12.86	13.76	16.35	11.68	7.61
2007	14.1	13.58	15.54	11.94	7.73
2008	13.5	14.15	16.57	12.62	8.04
2009	12.91	14.8	16.05	13.22	8.3
2010	16.31	14.64	16.7	13.28	8.55
2011	14.01	14.96	16.48	13.86	8.83
2012	14.94	15.43	16.52	14.12	9.22
2013	16.44	15.62	15.92	14.46	9.4
2014	14.98	15.29	15.92	14.53	9.54
2015	15.98	15.4	15.13	14.43	9.8
2016	17.27	16.01	14.64	14.85	9.84
2017	16.17	15.81	14.07	14.79	9.97
2018	17.53	16.06	13.65	14.34	10.04
2019	15.55	15.31	13.86	13.81	10.09
2020	17.81	15.39	13.54	13.46	10.21
2021	16.43	15.58	14.23	13.53	10.58
2022	15.76	15.56	13.05	13.32	10.52
2023	15.68	15.83	13.57	13.85	10.92

### 3.4 Region

From 1999-2013 (**Table 3**), it was determined this date range for the Western region to have an increase in AAMR, 2.60* (95% CI 2.32 to 3.0), and consequently the highest AAMR across all regions. The Southern region had 2 Jpoints, with the 2014–2023 year range having increased AAMR 0.87* (95% CI 0.36 to 1.92), and the second highest overall AAMR for all regions.

**Table 3:** Liver and IHBD cancer-related age-adjusted mortality rate per 100,000 people stratified by region, 1999-2023.

Year	Northeast	Midwest	South	West
1999	7.43	7.09	8.23	8.23
2000	7.65	6.98	7.91	8.67
2001	7.73	7.01	8.12	8.68
2002	7.75	7.28	8.37	9.07
2003	7.98	7.42	8.43	9.38
2004	8.29	7.51	8.56	9.44
2005	8.34	7.78	8.81	9.75
2006	8.61	7.98	8.95	9.58
2007	8.62	7.72	9.14	10.01
2008	9.02	8.12	9.56	10.29
2009	9.39	8.28	10.07	10.56
2010	9.61	8.86	10.02	10.83
2011	9.54	8.99	10.62	11.28
2012	10.06	9.56	10.81	11.81
2013	10.11	9.56	11.17	12.09
2014	10.2	9.79	11.62	11.62
2015	10.18	9.69	11.77	12.15
2016	10.45	10.22	11.76	12.12
2017	10.35	10.23	11.92	12.11
2018	9.95	10.42	11.9	12.31
2019	10.17	10.3	11.81	12.09
2020	10.11	10.45	11.99	12.02
2021	10.2	10.76	12.39	12.26
2022	10.03	10.61	12.22	12.27
2023	10.2	11	12.77	12.51

**Figure 5:**
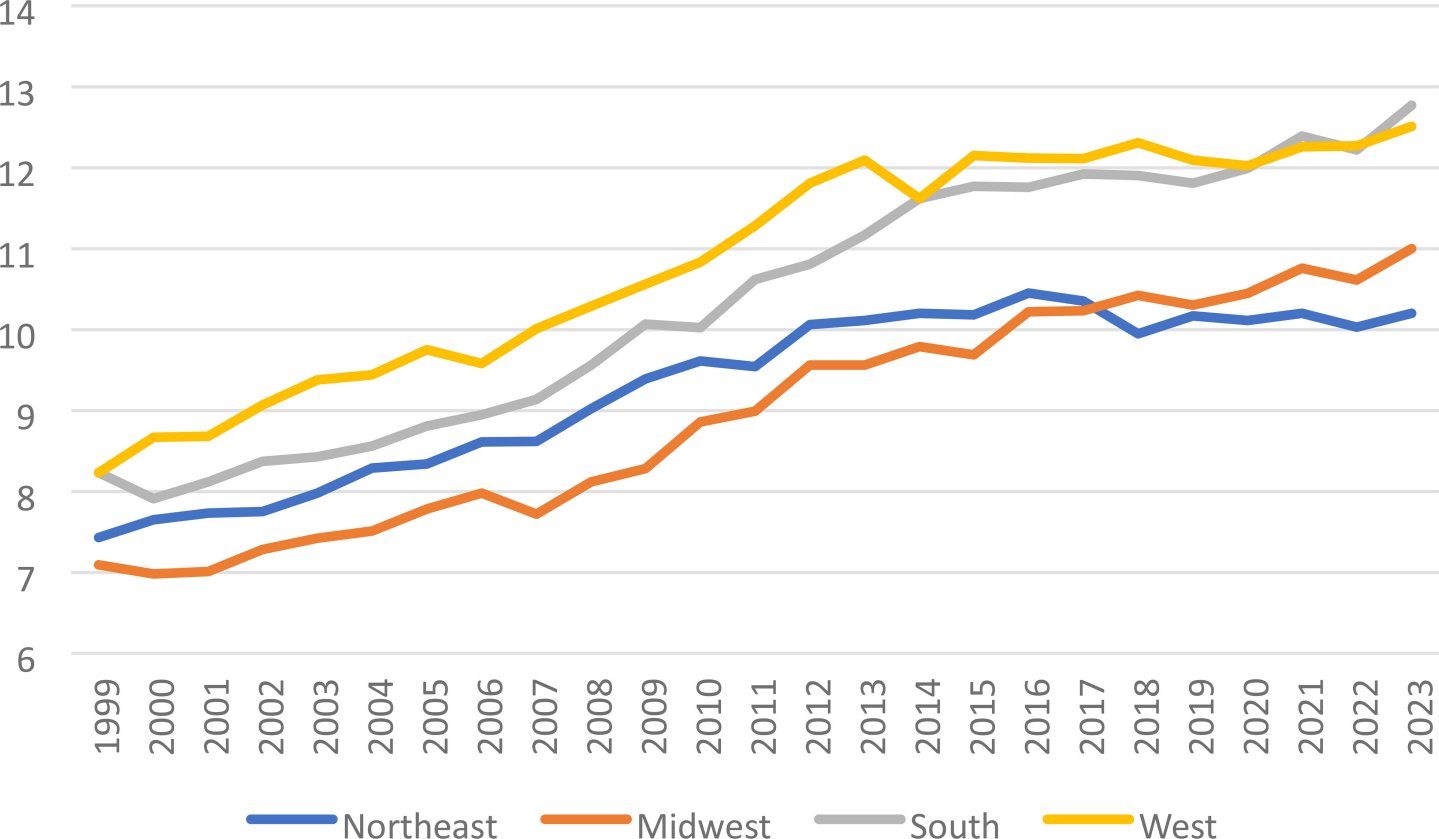
Liver and IHBD cancer-related age-adjusted mortality rate per 100,000 people; overall and stratified by region, 1999-2023.

**Figure 6:**
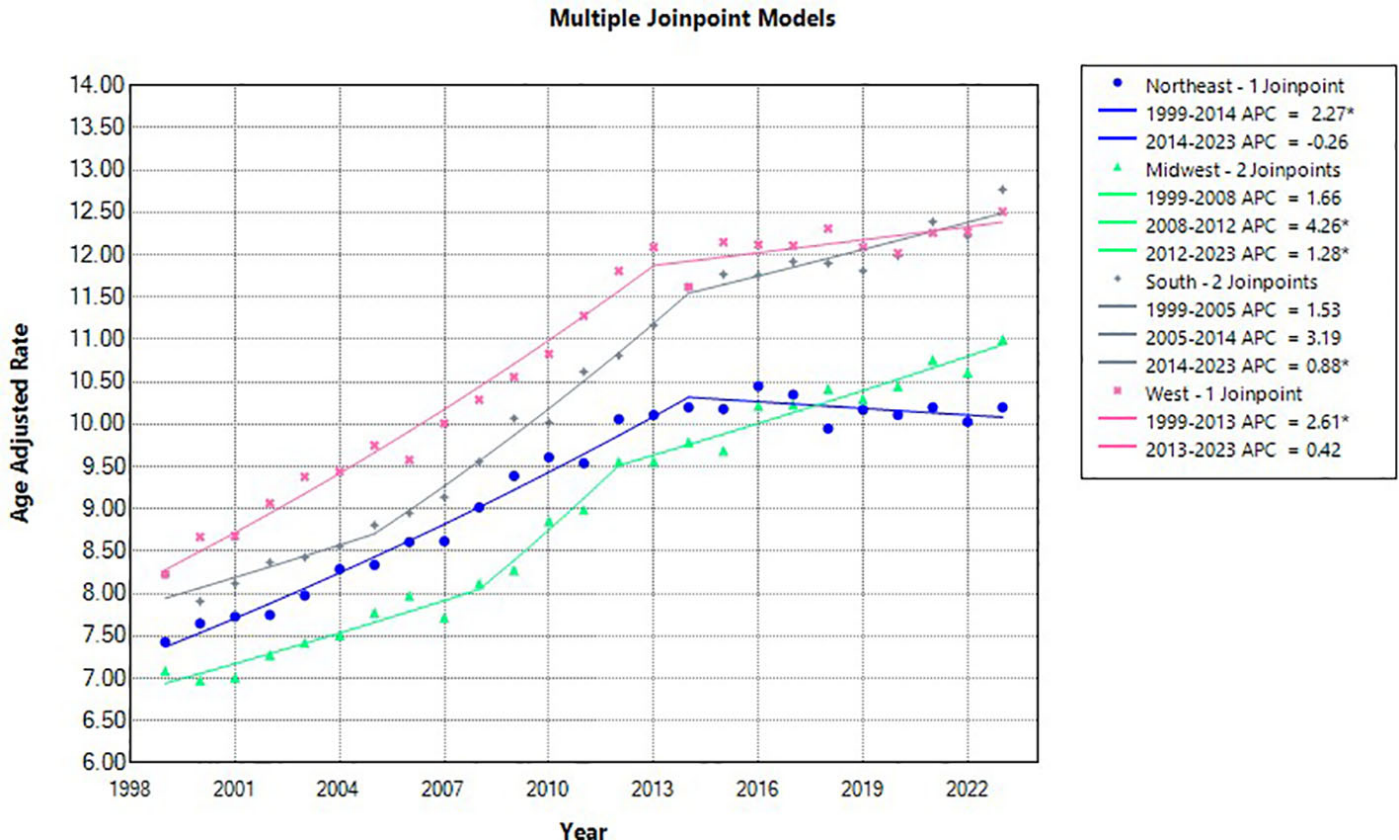
Joinpoint model of Liver and IHBD cancer-related AAMR per 100,000 people overall and stratified by region, 1999-2023 (*indicates the APC is statistically significant).

Subsequently, the year range 1999–2014 demonstrated a significant increase in AMMR for the Northeast region, 2.26* (95% CI 2.05 to 2.54), with no reported significant change in the year range 2015-2023. Finally, the Midwest region had the lowest overall AAMR for all regions, with 2 significant Jpoints, 2008-2012 4.26* (95% CI 2.78 to 5.86) and from 2012-2023 1.27* (95% CI 0.88 to 1.56). The AAPC was highest in the Midwestern region 1.91* (95% CI 1.75 to 2.06), followed by the South 1.90* (95% CI 1.72 to 22.15), then the West 1.68* (95% CI 1.54 to 1.86), and finally the lowest AAPC in the Northeast 1.31* (95% CI 1.18 to 1.44). (**Figures 5**, **6**).

### 3.5 10-year age groups

**Figure 7:**
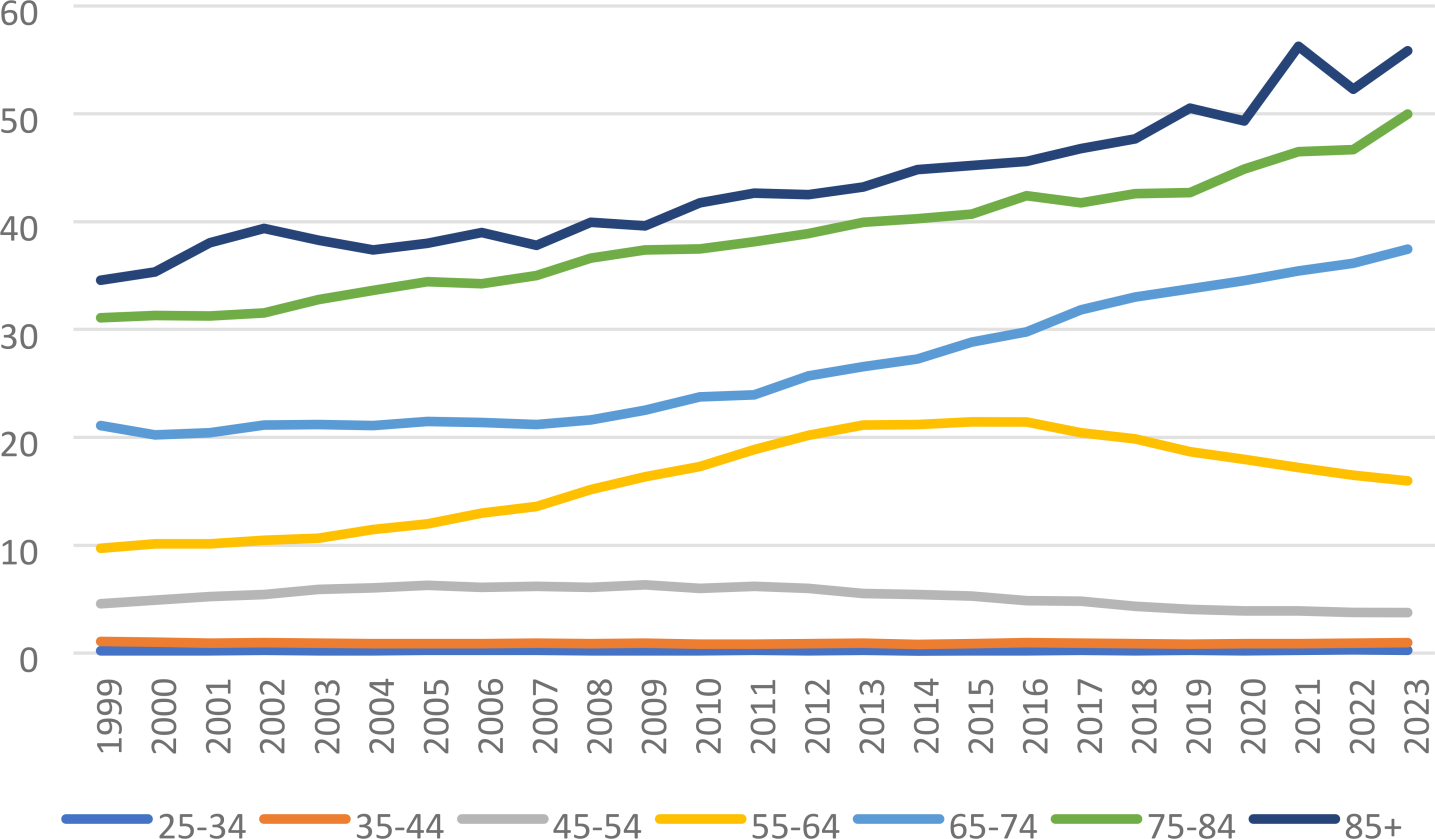
Liver and IHBD cancer-related age-adjusted mortality rate per 100,000 people; overall and stratified by 10-year-age groups, 1999–2023.

**Figure 8:**
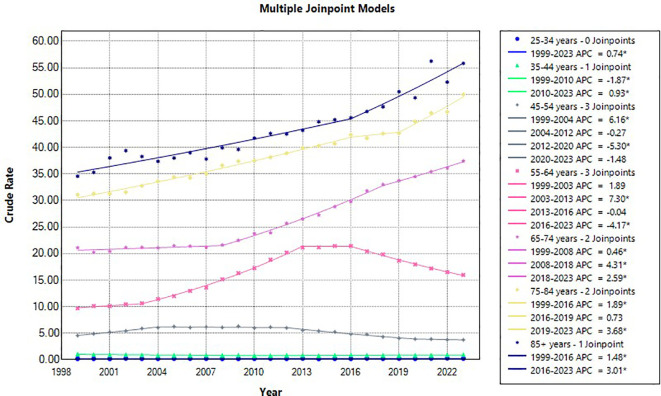
Joinpoint model of Liver and IHBD cancer-related AAMR per 100,000 people overall and stratified by 10-year-age groups, 1999-2023 (*indicates the APC is statistically significant).

Data was also divided into 10-year-age groups, excluding the age groups of <25 years due to a paucity of data (**Table 4**) Patients who are 85 years of age or older had the highest crude mortality rate for the entire study period (1999-2023), from 1999-2016 1.48* (95% CI 0.36 to 1.78) which increased to 3.00* (95% CI 2.12 to 5.81) for the following years 2016-2023. The next highest mortality rates were found in the 75–84 year old age range with 2 significant year ranges, 1999-2016 1.88* (95% CI 1.28 to 4.99), increasing to 3.67* (95% CI 2.22 to 6.10) during the 2019–2023 year range. The 65–74-year-old age range demonstrated the next highest mortality rates for the 3-year ranges: 1999-2008 0.46* (95% CI 0.03 to 0.80), 2008–2018 increasing to 4.31* (95% CI 4.08 to 4.66), and 2018-2023 2.59* (95% CI 1.99 to 3.04). The 55–64-year-old age group demonstrated 2 significant year ranges, 2003-2013 7.30* (95% CI 7.03 to 7.78) and the 2016–2023-year range decreased mortality rates -4.17* (95% CI -4.63 to -3.86). The 45–54 year age grouping also had 2 significant year ranges, 1999-2004 6.15* (95% CI 4.80 to 7.86) and the 2012–2020 year range which had a decrease in mortality: -5.29 (95% CI -6.92 to -2.64). The 35–44 year old age range had 2 significant year ranges with respect to mortality, 1999–2010 showed decreased mortality: -1.86* (95% CI -5.91 to -0.78) and the an increase in mortality from 2010-2023: 0.93* (95% CI 0.06 to 4.51). Finally, the 25–34 year old age range demonstrated significance in morality across the entire study 1999-2023, 0.74* (95% CI 0.14 to 1.38) (**Figures 4**, **7**). The 35–44 year age group demonstrated no significance with respect to AAPC, the 45–54 age group demonstrated decreased AAPC significantly: -0.84* (95% CI -1.11 to -0.60), with all other age ranges demonstrating significantly increased AAPC (**Figures 7**, **8**).

**Table 4:** Liver and IHBD cancer-related age-adjusted mortality rate per 100,000 people stratified by 10 year age groups, 1999-2023.

Year	25-34	35-44	45-54	55-64	65-74	75-84	85+
1999	0.21	1.08	4.57	9.72	21.12	31.09	34.57
2000	0.22	1.01	4.9	10.13	20.23	31.29	35.33
2001	0.2	0.94	5.23	10.13	20.41	31.27	38.03
2002	0.27	0.98	5.42	10.47	21.13	31.56	39.39
2003	0.23	0.94	5.91	10.66	21.19	32.78	38.29
2004	0.22	0.87	6.06	11.46	21.09	33.63	37.37
2005	0.24	0.88	6.27	12	21.48	34.42	38.01
2006	0.24	0.89	6.08	12.99	21.4	34.27	39.01
2007	0.24	0.91	6.19	13.61	21.2	35.01	37.82
2008	0.21	0.87	6.11	15.16	21.64	36.62	39.94
2009	0.21	0.9	6.33	16.34	22.51	37.4	39.63
2010	0.23	0.81	6	17.28	23.77	37.49	41.76
2011	0.25	0.82	6.17	18.86	23.93	38.13	42.63
2012	0.22	0.89	6.02	20.2	25.72	38.91	42.52
2013	0.27	0.9	5.55	21.14	26.58	39.93	43.22
2014	0.19	0.8	5.43	21.18	27.27	40.28	44.84
2015	0.23	0.88	5.27	21.44	28.84	40.7	45.22
2016	0.22	0.95	4.84	21.43	29.8	42.39	45.59
2017	0.27	0.9	4.8	20.44	31.83	41.75	46.79
2018	0.23	0.89	4.33	19.86	33.01	42.62	47.66
2019	0.26	0.84	4.05	18.66	33.76	42.68	50.51
2020	0.23	0.87	3.92	17.98	34.52	44.88	49.34
2021	0.27	0.89	3.91	17.21	35.43	46.49	56.26
2022	0.3	0.94	3.78	16.5	36.15	46.7	52.3
2023	0.26	0.98	3.76	15.99	37.45	49.98	55.84

## 4 Discussion

For this study period, the AAMR and APC increased significantly across several year periods. Most likely a large contributing factor for these trends are the increase in alcoholic liver disease, metastatic cancers to the liver, chronic hepatitis C infection, and metabolic dysfunction associated steatotic liver disease (MASLD) (14, 15). Furthermore, liver and intrahepatic bile duct cancer related deaths have significantly increased across the entire study period, with males being disproportionally affected compared to females; however, both sexes demonstrated increased mortality rates across the entire study period. Overall, the most likely explanation for increased overall incidence of these types of cancers in both men and women in recent years is strongly explained by the current US obesity epidemic (16). Liver and intrahepatic biliary cancers, especially hepatocellular carcinoma, are now known to be majorly caused by MASLD (17). Verily, regardless of sex, all the strong risk factors for these cancers are highly modifiable (e.g; low BMI, smoking cessation, limiting alcohol use, and vaccination protocols), fundamentally altering behaviors have the potential to drastically decrease risk across all genders.

One of the most surprising findings across the 1999–2023 study period was the significant decrease in liver and intrahepatic bile duct cancers in Asian and Pacific Islanders. In 1999 this racial group represented the highest AAMR across all races; by 2023 only white non-Hispanics had lower rates of Liver and intrahepatic bile duct caners. A change that was documented in the later part of the 1990s and early 2000s was community outreach in Asian America and Pacific Islander populations, this racial group represented at the time (1999) approximately over half of all chronic Hepatitis B infections in the US (a risk factor for these cancer types) (18). At the beginning of this study period groups began community outreach on the necessity for testing and vaccination for this virus, perhaps largely contributing to the significant fall in AAMR for these cancers across our entire study period in Asians and Pacific Islanders (19, 20). The rising incidence of hepatic and bile duct cancers among Hispanic/Latinos, Native Americans, and Caucasians is linked to shifts in disease epidemiology. Historically, viral hepatitis was a major driver of disease across all groups, however recent studies are concluding that a growing contribution is due to MASLD and to a lesser extent alcoholic liver disease (ALD) and the intersection of both. Hispanic/Latinos and Native American populations have an increased prevalence of MASLD due to obesity, diabetes, and to a lesser extent a genetic susceptibility (e.g., PNPLA3 genetic variants); this is compounded by higher rates of ALD that ultimately creates a 2 hit hypothesis as the cause of the increased cancers rate(s) among these ethnicities (21). While in Caucasian populations with HCC and intrahepatic bile duct malignancies, the increased rate is due to similar factors such as MASLD and ALD, but also due to overall aging cohorts and prior hepatitis C exposure (21). Research has demonstrated 2 major factors for poorly treated chronic conditions in all other races compared to white non-Hispanics: decreased Primary Care Physician (PCP) access and distrust of the medical system. Studies show that among non-white-non-Hispanics, there is decreased access to PCP in the United States (22) with an increase in HCC risk. Additionally, research has demonstrated that there is an inherent distrust in the healthcare system among non-white-non-Hispanic ethnics groups (23). Therapeutic alliance, and trust in the healthcare provider verily makes the patient more likely to have annual adult health maintenance screenings, be compliant with treatment regimens, and lifestyle change recommendations.

Easily apparent is the direct relationship to age and mortality risk of liver and intrahepatic bile duct cancers. From beginning to end of the study the 85+ year-old age group by far had the highest mortality rates. Most likely this represents better quality screening combined with the previously mentioned significant risk factors for these types of cancers (e.g.; obesity, diabetes, alcohol, cigarettes, and viral hepatitis) (24). These age groups were from the generations that had the highest cigarette smoking rates, and up until the 1980s was a quite commonly used drug (25). Fast forward 40 years and these individuals that possibly smoked for decades prior to quitting may have increased their risk for these cancers later in life when aging and accumulated gene mutations and shortening of telomeres have its greatest effect, verily increasing cancer risks (26). The same concept for obesity and diabetes explains why there are significantly increasing trends in AAMR for liver and intrahepatic bile duct cancers in the older age groups, years exposed to diabetes and obesity cause changes in the tissues of the hepatobiliary system leading to mutations and eventual increased cancers risk (27). It is also worth mentioning, that this age group did not have access to hepatitis vaccines until 1981 in the United States putting this group of people at increased risk of contracting this virus and increasing their risk for eventual liver and intrahepatic bile duct neoplasms (28).

All regions across the entire study period demonstrated a significant increase in AAMR from 1999-2023. The West and South regions showed higher rates of AAMR for Liver and Intrahepatic bile duct malignancies. Part of this may be explained by population race dynamics (29); the West and South have a higher percentage of Hispanic and Latino, Black, and, and Native American or Native Alaskan populations. These are already demonstrated in this study as having higher AAMR compared to white non-Hispanics, with a greater portion of white non-Hispanics population densities in the Midwest and Northeast possibly explain the lower AAMR in those regions compared to the West and South. Census information also confirms higher rates of poverty at a regional level comparable to the data from our study range (30), with least to highest poverty rates by region as follows Northwest<Midwest<West<South (31); this directly correlates to AAMR of Liver and intrahepatic bile duct cancers in our study by regional prevalence. With respect to primary liver cancers, there is a demonstrated a link between overall poverty levels and increased rates of these cancers, with higher rates in Texas and Louisiana in one study (32), both found in the South region (33). Invariably, across the entire country, poverty increases mortality risk for all cancers, and specifically liver and intrahepatic bile duct cancers (34). Furthermore, it is established that overall rural counties have a paucity of access to quality healthcare and increased rates of morbidity and mortality in the US (35).

## 5 Limitations

A primary limitation of this study would be the inability using the CDC database to exclude underlying liver disease data (e.g., primary sclerosing cholangitis, MASH, ALD, etc.) from the patient population as past medical history of each data point is not accessible. Additionally, this study was not able to differentiate between intrahepatic and bile duct cancers, so we did not examine these trends separately. Furthermore, although treatments are limited for intrahepatic and bile duct cancers, the treatments (e.g, chemotherapy, immunotherapy, surgery, endoscopic intervention) may affect overall outcome and mortality. Another weakness with respect to liver and intrahepatic bile duct cancers is screening, many of these malignancies are diagnosed late stage, well past when treatment modalities are most efficacious. To date there is no recommend ideal screening time frame for these cancers, unlike colonoscopy regimens for colorectal cancers, smoking history and annual low dose CT scans for lung cancers, and pap smears for cervical cancers (USPSTF); all of which have much evidence to support exact ideal screening guidelines, time frames, and modalities (36). Furthermore, even though age and obesity are significant risk factors for these cancers, no research to date supports screening these populations at any regular interval. Perhaps this is where funding should be focused at a large scale, huge cohorts of individuals fitting these parameters should have annual low dose abdominal CT scans plus or minus markers such as Alpha-fetoprotein, carbohydrate antigen 19-9, and/or various other tumor markers may help delineate better guidelines.

## 6 Conclusion

This study supports that at a national level, across multiple demographics, the trends in AAMR for liver and intrahepatic bile duct cancers are on the rise. Significant differences in AAMR were found among gender (male > female), race (decreased AAMR in Asian and Pacific islanders, increased in all other races, white non-Hispanic < all other races), region (South > West > Midwest > Northwest), and that liver and bile duct cancer AAMR is directly proportionate to increasing age group. Focused efforts to decrease AAMR for these cancers need to focus on racial and geographic disparities, taking in to account access to limited health care providers in certain areas, and higher rates of poverty in certain regions. Numerous road blocks still exist to address these issues (e.g.; limited PCP, cost of screening exams, late staging of cancers at diagnosis, and best practice for screening for these cancers), and future endeavors should attribute funding to amend these disparities.

## Data Availability

The original contributions presented in the study are included in the article/supplementary material. Further inquiries can be directed to the corresponding author.
